# Anti-SARS-Cov-2 S-RBD IgG Formed after BNT162b2 Vaccination Can Bind C1q and Activate Complement

**DOI:** 10.1155/2022/7263740

**Published:** 2022-12-17

**Authors:** Anas H. A. Abu-Humaidan, Fatima M. Ahmad, Dima Awajan, Raba'a F. Jarrar, Nader Alaridah

**Affiliations:** ^1^Department of Pathology, Microbiology and Forensic Medicine, School of Medicine, The University of Jordan, Amman 11942, Jordan; ^2^Department of the Clinical Laboratory Sciences, School of Science, The University of Jordan, Amman 11942, Jordan; ^3^Department of Clinical Pharmacy and Therapeutics, Applied Science Private University, Amman 11931, Jordan

## Abstract

**Background:**

The ability of vaccine-induced antibodies to bind C1q could affect pathogen neutralization. In this study, we investigated C1q binding and subsequent complement activation by anti-spike (S) protein receptor-binding domain (RBD) specific antibodies produced following vaccination with either the mRNA vaccine BNT162b2 or the inactivated vaccine BBIBP-CorV.

**Methods:**

Serum samples were collected in the period of July 2021-March 2022. Participants' demographic data, type of vaccine, date of vaccination, as well as adverse effects of the vaccine were recorded. The serum samples were incubated with S protein RBD-coated plates. Levels of human IgG, IgA, IgM, C1q, and mannose-binding lectin (MBL) that were bound to the plate, as well as formed C3d, and C5b-9 were compared between different groups of participants.

**Results:**

A total of 151 samples were collected from vaccinated (*n* = 116) and nonvaccinated (*n* = 35) participants. Participants who received either one or two doses of BNT162b2 formed higher levels of anti-RBD IgG and IgA than participants who received BBIBP-CorV. The anti-RBD IgG formed following either vaccine bound C1q, but significantly more C1q binding was observed in participants who received BNT162b2. Subsequently, C5b-9 formation was significantly higher in participants who received BNT162b2, while no significant difference in C5b-9 formation was found between the nonvaccinated and BBIBP-CorV groups. The formation of C5b-9 was strongly correlated to C1q binding and not to MBL binding, additionally, the ratio of formed C5b-9/bound C1q was significantly higher in the BNT162b2 group.

**Conclusion:**

Anti-RBD IgG formed following vaccination can bind C1q with subsequent complement activation, and the degree of terminal complement pathway activation differed between vaccines, which could play a role in the protection offered by COVID-19 vaccines. Further investigation into the correlation between vaccine protection and vaccine-induced antibodies' ability to activate complement is required.

## 1. Introduction

COVID-19 caused by the severe acute respiratory syndrome coronavirus 2 (SARS-COV-2) was declared a pandemic in 2020 and has since infected more than 600 million people and resulted in over 6.5 million deaths. Vaccines produced against SARS-COV-2 had a major role in limiting the spread of infection and decreasing hospitalization [1]. There are currently over 150 vaccines in clinical development with around 10 vaccines approved for use, among them were the mRNA BNT162b2 developed by Pfizer–BioNTech and the inactivated vaccine BBIBP-CorV developed by Sinopharm. The two types of vaccines were found to offer various degrees of protection from COVID-19 in terms of mortality and hospitalization [2, 3] and varying humoral and T-cell-mediated immune responses [4]. Those vaccines were the two main vaccines approved for use in Jordan in addition to the nonreplicating viral vector Oxford/AstraZeneca vaccine. According to recent statistics, nearly half of the population received at least 1 dose of approved COVID-19 vaccines (retrieved from https://coronavirus.jhu.edu/region/jordan).

Vaccines initiate the formation of antibodies, prime T-cell-mediated immunity, and establish memory B- and T-cell populations [5]. The protection offered by antibodies can be ascribed to several effector mechanisms; one of which is binding to antigens on the pathogen's surface, thereby neutralizing its ability to bind to host cells [6]. Several COVID-19 vaccines targeted the Spike (S) protein in SARS-COV-2, which is important for viral attachment to host angiotensin-converting enzyme 2 (ACE2) and entry into host cells [7–9]. Some of the antibodies produced following vaccination against SARS-COV-2 bind the receptor-binding domain of the S protein (RBD); such antibodies are commonly referred to as neutralizing antibodies because they target an essential domain for the establishment of infection [10].

In addition to binding viral antigens through the Fab portion, the Fc portion of antibodies can interact with Fc receptors found on immune cells [11] and with C1q of the complement system [12]. The complement system is made of several proteins that are found in tissue and circulation; those proteins are activated in a cascade-like manner to yield fragments that propagate the immune response [13]. Pattern recognition molecules such as C1q and mannose-binding lectin (MBL) can opsonize pathogens and activate the classical and lectin pathways, respectively [14]. Subsequently, C4 and C2 are cleaved to form the C3 convertase, which cleaves C3 and leads to the formation of the opsonin C3b as well as the anaphylatoxin C3a, which interacts with a variety of innate and adaptive immune cells [15]. Finally, activation of the terminal pathway through C5 cleavage leads to the formation of another potent anaphylatoxin, C5a, and leads to C5b deposition on the pathogen surface, which initiates the formation of the pore-forming complex C5b-9 [16].

In COVID-19, activation of complement can occur through the classical, lectin, and alternative pathways [17]. Activation of complement is meant to be a protective immune response against SARS-COV-2, but aberrant activation of complement is thought to contribute to the deterioration of patients with severe disease, as evidenced by the elevated levels of complement activation fragments found in the sera and lungs of hospitalized patients [18].

While the role of complement in COVID-19 was investigated in several studies, less attention has been given to the role of complement in the protection offered by COVID-19 vaccines [19]. As mentioned above, C1q can bind immune complexes (antibodies bound to their respective antigen) and activate the classical pathway of complement, but the level of C1q binding and complement activation can vary between antibodies, for example, a recent study indicated that the structural features, such as the glycosylation patterns of the Fc portion of IgG could affect FcɣR and C1q binding [20]. Therefore, this study aimed to assess C1q binding and subsequent complement activation by anti-RBD antibodies produced following vaccination with either the mRNA vaccine BNT162b2 or the inactivated vaccine BBIBP-CorV.

## 2. Materials and Methods

### 2.1. Study Design and Population

This study was conducted from July 2021 to March 2022. The participants were recruited at the vaccination centers at the University of Jordan before receiving either their first, second, or booster vaccine doses. The vaccines included in the study were the inactivated vaccine BBIBP-CorV (Sinopharm) and the mRNA vaccine BNT162b2 (Pfizer-BioNTech), which were the two main vaccines provided in Jordan. The records for COVID-19 vaccination in Jordan were electronic, each participant received a text message of the date and type of vaccine they had, which allowed for accurate documentation of vaccine data. For each participant, the following data was recorded: (1) demographics included age, sex, height, and weight. (2) Vaccination date, type, and doses. (3) Adverse events following vaccination.

The participants were mainly residents of the capital Amman, mostly Jordanian, and all were from the Middle East and North Africa region (MENA). The inclusion criterion was age ≥ 18 years of age. While exclusion criteria were (1) having used complement inhibition therapy within the last 3 months and (2) documented complement deficiency or other immunodeficiencies.

### 2.2. Sample Collection

Samples were collected in plain blood collection tubes with a gel separator, then immediately preserved at 4°C and allowed to clot for a maximum of 2 hours. Samples were then transported on ice for further processing. Serum was collected by centrifugation of clotted blood tubes for 15 minutes at 1500 × g and 4°C. The serum was directly aliquoted into sterile EP tubes and stored immediately at -80°C until analysis. Serum samples were thawed on ice on the day of the experiments. Repeat freeze-thaw cycles were avoided to prevent protein degradation.

### 2.3. Relative Quantification of anti-RBD Immunoglobulins and Complement Proteins

The assay used for relative quantification of IgG, IgA, IgM, C1q, C3d, and C5b-9 employed an indirect sandwich enzyme-linked immunosorbent assay (ELISA), using 96-Well SARS-CoV-2 Spike protein RBD-Coated Plates (ACRO Biosystems, cat. Number RP-13-21 AU-21 AU), which were already blocked with 2% Bovine Serum Albumin (BSA). The plates and serum from participants were brought to room temperature, then each well received 100 *μ*l from a solution of 1% serum in phosphate-buffered saline with 0.1% Tween 20 (PBST) (Thermo Scientific, cat. Number 28352) and incubated for 90 mins at 37°C. Some wells were incubated with PBST only and were used as controls. Plates were then washed three times with PBST and blocked with 120 *μ*l of blocking buffer made of 2% BSA (Thermo Scientific, cat. Number 37525) in PBST for 30 min at 37°C.

For IgG, IgA, and IgM measurements, plates were incubated with either horseradish peroxidase (HRP)-conjugated rabbit anti-human IgG polyclonal antibodies (Abcam, cat. Number ab6759), horseradish peroxidase (HRP)-conjugated rabbit anti-human IgA polyclonal antibodies (Novus Biologicals, cat. Number NBP1-74913), and HRP-conjugated rabbit anti-human IgM polyclonal antibodies (Novus Biologicals, cat. Number NBP1-75081), respectively, both at a concentration of 1 : 2000 in blocking buffer at 4°C overnight.

While for C1q, MBL, C3d, and C5b-9 measurement, the wells were washed three times with PBST, then incubated with either rabbit anti-human C1q polyclonal antibodies (MyBioSource, cat. Number MBS573500), rabbit anti-human MBL polyclonal antibodies (MyBioSource, cat. Number MBS2003693), rabbit anti-human C3d monoclonal antibodies (Novus Biologicals, cat. Number NBP1-79074), and mouse anti-human C5b-9 monoclonal antibodies (Novus Biologicals, cat. Number NBP1-05120), respectively, at a concentration of 1 : 500 in blocking buffer at 4°C overnight. The next day, the wells were washed three times with PBST, then incubated with the following secondary antibodies for 3 hours at room temperature; HRP-conjugated goat anti-rabbit polyclonal antibodies (Novex by Life Technologies, cat. Number 90-11-110520) for C1q, MBL, and C3d, or HRP-conjugated goat anti-mouse polyclonal antibodies (Abcam, cat. Number ab97245) for C5b-9.

After the incubation with HRP-conjugated antibodies, all plates were washed three times with PBST. The chemiluminescence signal in the plates was measured as previously described [21], briefly, 50 *μ*l of the enhanced chemiluminescence (ECL) substrate (Promega, cat. Number W1015) was added to each well for 5 minutes, after the development of the signal images of the plates were taken using Chemidoc (Bio-Rad). The image files were analysed using Fiji/ImageJ [22]. The chemiluminescence signal from wells incubated with 1% serum (S) was divided by the noise from the control wells incubated with PBST (N) and was presented in the figures as (S/N).

### 2.4. Ethical Approval

The Institutional Review Board (IRB) approved the study protocol at UJ (Ref. No. 68/2021). In addition, the work was conducted according to the principles of Good Clinical Practice (GCP) that has its origin in the Declaration of Helsinki (64th World Medical Association General Assembly, Fortaleza, Brazil, October 2013). All collected data were treated with confidentiality. Participation in the study was voluntary. A written and signed informed consent was obtained from all participants who agreed to participate following a full explanation of the study objectives.

### 2.5. Data Analysis

The data generated was organized in Microsoft Excel, and statistical analysis was carried out using GraphPad Prism 8 software for analysis. Results were presented as (mean ± SD) unless stated otherwise. The Shapiro-Wilk test was first used to test the distribution of data, subsequently, the nonparametric Mann–Whitney *U* test was used for single pairwise comparisons between dose-matched vaccine groups, while the nonparametric Kruskal–Wallis test followed by Dunn's test was used for multiple pairwise comparisons between vaccine groups [23]. Wilcoxon matched-pairs signed rank test was used when comparing data from participants before and after vaccination. The nonparametric Spearman correlation coefficient was used to denote the magnitude and direction of correlation between different variables. All statistical tests were two-tailed and a probability value (*P*) less than 0.05 was considered significant.

## 3. Results

### 3.1. Demographics and Vaccine-Related Information

All participants were above 18 years of age, and none reported any health conditions. We divided the participants who did not receive a vaccine into two groups: those who either tested negative for COVID-19 using a PCR test or did not perform a PCR test and did not report any COVID-19 symptoms at any time before sample collection (*unvaccinated*) (*n* = 22), and those who tested positive for COVID-19 using a PCR test at any time before sample collection (PCR (+)) (*n* = 13). (Table 1).

While participants who were vaccinated were divided by the type of vaccine and the number of doses they received into the following groups: one dose of the BNT162b2 vaccine (1DP) (*n* = 29), two doses of the BNT162b2 vaccine (2DP) (*n* = 27), one dose of the BBIBP-CorV vaccine (1DS) (*n* = 21), two doses of the BBIBP-CorV vaccine (2DS) (*n* = 24), and a third (booster) dose of BNT162b2 vaccine following two doses of either the BBIBP-CorV or BNT162b2 vaccine (3D) (*n* = 15) (Table 1).

### 3.2. Relative Levels of anti-RBD IgG, IgA, and IgM

**Figure 1:**
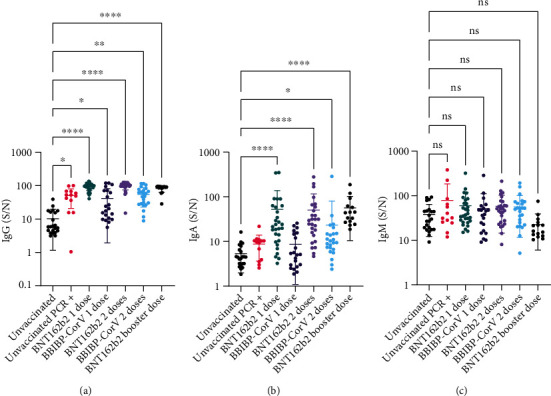
Measurement of anti-RBD IgG, IgA, and IgM. Participants were divided by their vaccination status, type of vaccine, and dose of vaccine. Serum samples were incubated with RBD-coated plates; subsequently, HRP-tagged anti-human IgG, IgA, or IgM antibodies were used to detect the presence of bound IgG, IgA, and IgM, respectively. (S/N) represents the chemiluminescence signal generated from wells incubated with 1% serum divided by the signal in control wells incubated with PBST. (a) Represents anti-RBD IgG levels while (b) represents anti-RBD IgA levels, and (c) represents anti-RBD IgM levels. Bold horizontal lines represent the mean of each group, while whiskers represent the standard deviation. Some error bars were clipped at the axis limit. Dunn's multiple comparisons statistical test following Kruskal–Wallis test was used to compare various groups to the *unvaccinated* group. ns *P* > 0.05, ^∗^*P* ≤ 0.05, ^∗∗^*P* ≤ 0.01, ^∗∗∗∗^*P* ≤ 0.0001.

Analysis of relative anti-RBD IgG levels in various groups of participants was done by comparing each group to the *unvaccinated* group as a control. Using Dunn's multiple comparisons statistical test following the Kruskal–Wallis test indicated that all groups had significantly higher anti-RBD IgG levels than the *unvaccinated* group (Figure 1(a)). To assess the difference in anti-RBD IgG formation between BNT162b2 and BBIBP-CorV vaccines, the relative level of anti-RBD IgG was compared in each vaccine group using the Mann–Whitney statistical test, the comparison revealed higher levels of anti-RBD IgG in the 1DP compared to the 1DS group (mean (S/N): 93.79 ± 22.17, vs. 41.85 ± 39.92, respectively, *P* < 0.0001) as well as higher levels in the 2DP compared to the 2DS group (mean (S/N): 99.27 ± 24.84, vs. 54.91 ± 31.90, respectively, *P* < 0.0001). There was no significant difference between one or two doses of either vaccine.

Similar results to anti-RBD IgG were obtained with anti-RBD IgA, although the groups PCR (+) and 1DS had higher mean levels than the *unvaccinated* group, it was not statistically significant (Figure 1(b)). Using the Mann–Whitney statistical test, levels of anti-RBD IgA were higher in the 1DP compared to the 1DS group (mean (S/N): 52.18 ± 85.88 vs. 8.65 ± 7.56, respectively, *P* < 0.0001) as well as higher levels in the 2DP compared to the 2DS group (mean (S/N): 50.69 ± 65.17, vs. 23.44 ± 56.81, respectively, *P* < 0.0001). While there was no difference between the 1DP and 2DP groups, the 2DS had significantly higher IgA than the 1DS group (*P* = 0.0344).

The same statistical analysis was used to compare the groups of participants in terms of anti-RBD IgM levels, there was no significant difference in any of the groups when compared to the *unvaccinated* group (Figure 1(c)).

Due to the setup of this study, the time between the latest vaccine dose and sample collection varied among participants, so we assessed the correlation between the time since the latest vaccine dose and the levels of anti-RBD IgG, IgA, and IgM in vaccinated participants, we found a negative and weak yet statistically significant correlation using Spearman's correlation coefficient (rs) in IgG and IgA but not IgM (Supplementary Figure 1).

In aggregate, we confirmed that participants with a previous natural infection or vaccination with either dose of BNT162b2 or BBIBP-CorV had increased anti-RBD IgG and IgA. The anti-RBD IgG and IgA levels were significantly higher in the BNT162b2 vaccine groups. In addition, a significant difference in anti-RBD IgM was not found in any of the groups.

### 3.3. C1q and MBL Binding

**Figure 2:**
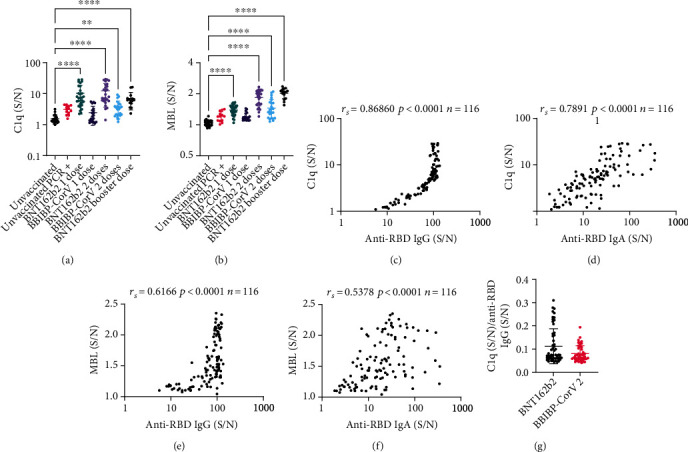
Measurement of C1q and MBL binding and their correlation to immunoglobulins. Participants were divided by their vaccination status, type of vaccine, and dose of vaccine. Serum samples were incubated with RBD-coated plates; bound C1q was subsequently measured using an indirect sandwich immunoassay. (S/N) represents the chemiluminescence signal generated from wells incubated with 1% serum divided by the signal in control wells incubated with PBST. (a) Represents bound C1q levels while (b) represents bound MBL levels. (c) A log-log scatter plot that correlates C1q to anti-RBD IgG and (d) a log-log scatter plot that correlates C1q to anti-RBD IgA levels in all vaccinated participants. (e) and (f) are scatter plots that correlate MBL to anti-RBD IgG and IgA levels, respectively, in all vaccinated participants. Spearman's correlation coefficient (*r*_s_), *P* value, and the number of participants (*n*) are displayed on the plot. (g) Individual values of C1q (S/N) per anti-RBD IgG (S/N) in each participant were calculated and displayed in a scatter plot for the two types of vaccines. In scatter plots, each circle represents one participant, bold horizontal lines represent the mean of each group, while whiskers represent the standard deviation. Dunn's multiple comparisons statistical test following Kruskal–Wallis test was used to compare various groups to the *unvaccinated* group. Only significant pairwise comparisons are displayed, ^∗∗^*P* ≤ 0.01, ^∗∗∗∗^*P* ≤ 0.0001.

Analysis of differences in C1q and MBL binding among groups of participants was done using Dunn's multiple comparisons statistical test following Kruskal–Wallis test and indicated that the 1DP, 2DP, 2DS, and 3D groups had both, significantly higher bound C1q, as well as MBL, than the *unvaccinated* group (Figures 2(a) and 2(b)).

To assess the difference in C1q binding between BNT162b2 and BBIBP-CorV vaccines, the relative level of bound C1q was compared using the Mann–Whitney statistical test between each vaccine group, the comparison revealed higher levels of bound C1q in the 1DP compared to the 1DS group (mean (S/N): 10.27 ± 8.082, vs. 2.434 ± 1.496, respectively, *P* < 0.0001), as well as higher levels in the 2DP compared to the 2DS group (mean (S/N): 12.39 ± 8.767, vs. 3.622 ± 2.101, respectively, *P* < 0.0001). There was no significant difference between one or two doses of either vaccine. An assessment of MBL binding also revealed more MBL binding in the 1DP compared to the 1DS group (mean (S/N): 1.389 ± 0.161, vs. 1.195 ± 0.105, respectively, *P* < 0.0001), as well as in the 2DP compared to the 2DS group (mean (S/N): 1.836 ± 0.314, vs. 1.450 ± 0.307, respectively, *P* < 0.0001). But unlike C1q, there was also more binding in the 2DP compared to the 1DP (*P* < 0.0001) and the 2DS compared to the 1DS group (*P* = 0.003), indicating increased MBL binding after the second dose of either vaccine.

To confirm that C1q was bound to the anti-RBD IgG, the correlation of bound C1q to IgG in all vaccinated participants (*n* = 116) was assessed using Spearman's correlation coefficient (rs). A strong and significant correlation was found between bound C1q and anti-RBD IgG (rs = 0.87, *P* < 0.0001) (Figure 2(c)). Notably, the correlation between C1q and anti-RBD IgG was not linear after a certain point in the range tested (Figure 2(c)). The correlation between C1q and IgA was assessed as well and demonstrated a strong significant correlation (rs = 0.79, *P* < 0.0001) (Figure 2(d)). Finally, we also investigated whether bound MBL was associated with IgG or IgA levels and found a moderate and significant correlation to IgG (rs = 0.62, *P* < 0.0001) and IgA levels (rs = 0.54, *P* < 0.0001) (Figures 2(e) and 2(f), respectively).

To address whether the increased binding of C1q in the BNT162b2 group was solely due to the increase in anti-RBD IgG level or due to the increased ability to bind C1q, we examined the ratio of C1q/anti-RBD IgG in each participant in the BNT162b2 and BBIBP-CorV vaccine groups (Figure 2(g)). We found no significant difference in the ratio between the 2 groups suggesting that the amount of C1q bound per IgG did not differ significantly.

Taken together, the data indicate that the anti-RBD IgG formed following vaccination with either BNT162b2 or BBIBP-CorV were able to bind C1q, and to a lesser extent, possibly MBL. Yet BNT162b2 led to the formation of more anti-RBD IgG and subsequently more C1q and MBL were bound.

### 3.4. Activation of Complement and Formation of C5b-9

**Figure 3:**
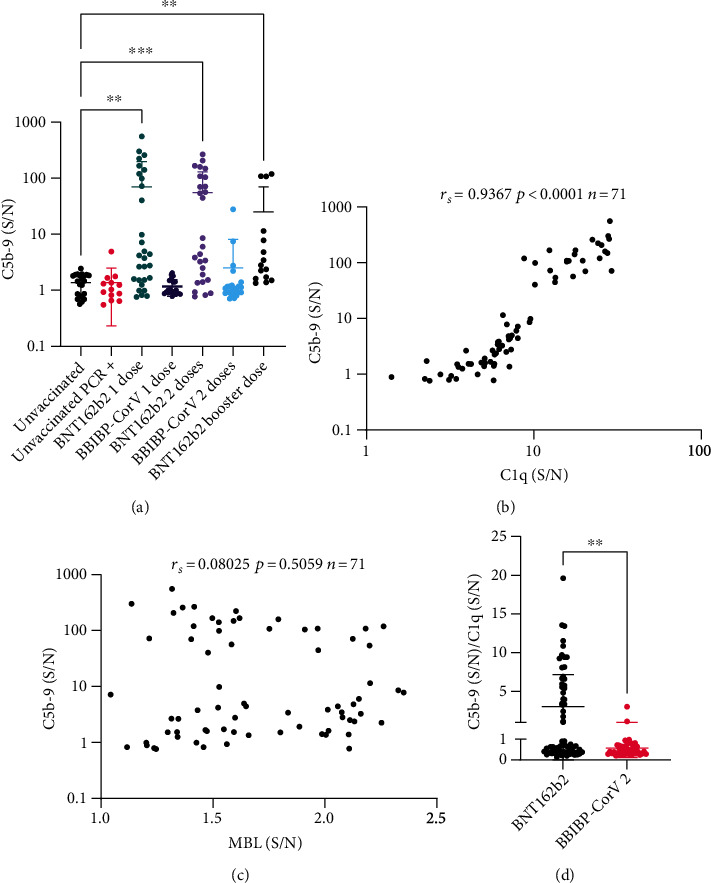
Measurement of C5b-9 formation. Participants were divided by their vaccination status, type of vaccine, and dose of vaccine. Serum samples were incubated with RBD-coated plates; the formation of C5b-9 was subsequently measured using an indirect immunoassay. (S/N) represents the chemiluminescence signal generated from wells incubated with 1% serum divided by the signal in control wells incubated with PBST. (a) Represents formed C5b-9 levels while (b) and (c) represents a scatter plot that correlates C5b-9 to C1q and MBL levels, respectively, in BNT162b2 vaccinated individuals, Spearman's correlation coefficient (r_s_), *P* value, and the number of participants (*n*) are displayed on the plot. (d) Individual values of C5b-9 per C1q in each participant were calculated and displayed in a scatter plot for the two types of vaccines. In scatter plots, each circle represents one participant, bold horizontal lines represent the mean of each group, while whiskers represent the standard deviation. Some error bars were clipped at the axis limit. Dunn's multiple comparisons statistical test following Kruskal–Wallis test was used to compare various groups to the *unvaccinated* group. Only significant pairwise comparisons are displayed, ^∗∗^*P* ≤ 0.01, ^∗∗∗^*P* ≤ 0.001.

To assess whether the binding of C1q or MBL led to activation of the terminal complement pathway, C5b-9 formation was used as an indicator. The analysis using Dunn's multiple comparisons statistical test following the Kruskal–Wallis test indicated that the 1DP, 2DP, and 3D groups formed significantly more C5b-9 than the *unvaccinated* group (Figure 3(a)). Notably, BNT162b2 vaccinated participants had a large range of terminal pathway activation, with a minimum, maximum, and mean fold change over control signal of 0.7593, 557.8, and 70.12, respectively, in the 1DP group, and a minimum, maximum, and mean of 0.7727, 265.3 in the 2DP group. To confirm activation of complement, measurement of C3d was performed as well in 47 participants and revealed a similar trend to C5b-9, but in addition to the 1DP, 2DP, and 3D groups, the 2DS group was also significantly higher than the *unvaccinated* group (Supplementary Figure 2).

The correlation between C1q and C5b-9 formation was assessed in BNT162b2 vaccinated groups (1DP, 2DP, 3D) (*n* = 71) since they were the only groups that showed significantly higher C5b-9 than the *unvaccinated* group. A very strong and significant correlation was found between bound C1q and C5b-9 (rs = 0.94, *P* < 0.0001) (Figure 3(b)). On the other hand, MBL showed no correlation to C5b-9 in the same group (*rs* = 0.10, *P* = 0.5059) (Figure 3(c)). This indicated that the classical pathway activation rather than the lectin pathway is closely associated with the activation of the terminal pathway and formation of C5b-9.

To address whether the increased C5b-9 formation in the BNT162b2 group was solely due to the increase in C1q binding or due to other factors, we examined the ratio of formed C5b-9/bound C1q in each participant in the BNT162b2 and BBIBP-CorV vaccine groups (Figure 3(d)). We found significantly higher C5b-9 formation per C1q binding in the BNT162b2 group, presumably due to a more stable C1q binding leading to increased complement activation.

### 3.5. Complement Activation before and after BNT162b2 Vaccination

To confirm the findings of increased anti-RBD IgG and IgA, increased binding of C1q, and increased complement activation following vaccination with the BNT162b2, we examined samples from 19 participants who provided a sample before and after the first dose of BNT162b2, the time between the dose and sample collection was a median of 23 days (95% CI 22-31). Additionally, 5 of the participants provided a second sample after the second dose, and the time between the dose and sample collection was a median of 19 days (95% CI 14-37).

**Figure 4:**
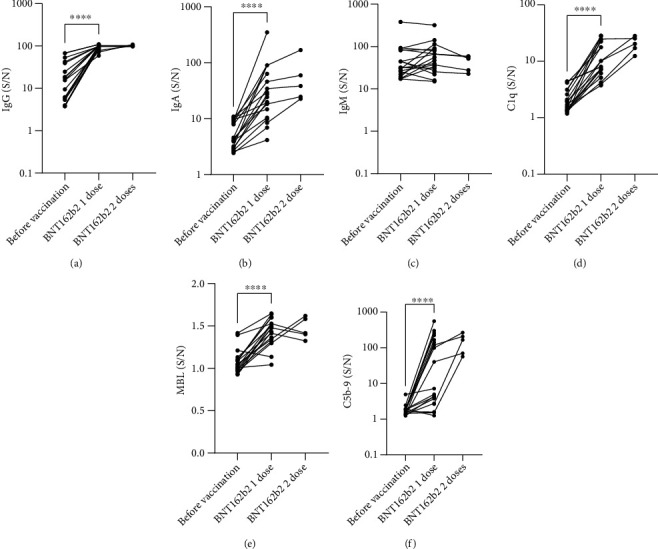
Complement activation before and after BNT162b2 vaccination. 19 participants provided samples before and after the first dose of BNT162b2, and 5 participants provided samples before and after the second dose of BNT162b2. Serum samples were incubated with RBD-coated plates (a) bound IgG, (b) IgA, (c) IgM, (d) C1q, (e) MBL, and (f) formed C5b-9 were subsequently measured as described in the methods section. (S/N) represents the chemiluminescence signal generated from wells incubated with 1% serum divided by the signal in control wells incubated with PBST. Each circle in the scatter plots represents one sample, and the circles connected by lines represent before and after samples from the same participant. Only significant pairwise comparisons are displayed, ^∗∗∗∗^*P* ≤ 0.0001.

Vaccination with one dose confirmed the significant increase in anti-RBD IgG and IgA but not IgM (Figures 4(a)–4(c)), in addition, bound C1q and MBL were increased (Figures 4(d) and 4(e)), and activation of the terminal pathway was increased as evident by C5b-9 formation (Figure 4(f)).

## 4. Discussion

Vaccination confers immunity through several mechanisms, one of which is antibody production. Antibodies produced against SARS-COV-2 RBD-S are often considered neutralizing due to their ability to inhibit viral binding and entry into host cells. But other effector functions of antibodies also contribute to conferring protection against infection, such as activation of complement through C1q binding. This study is aimed at assessing the ability of anti-RBD antibodies formed after vaccination with either BBIBP-CorV or BNT162b2 in fixing C1q and activating the complement system.

The assay we employed for relative quantification of antibodies and complement proteins used signal from PBST-treated wells as the background signal, and the chemiluminescent signal from wells treated with 1% serum as the true signal, the choice of 1% serum was guided by current literature and was confirmed through optimization assays. Although we cannot rule out the unspecific binding of IgG, IgA, and IgM especially since the signal in the *unvaccinated* group was significantly higher than the background, the group of *unvaccinated* participants served as the control group for all statistical analysis purposes in this study, effectively discounting the unspecific binding if present.

Since the participants were randomly recruited at vaccination centers, it was difficult to ascertain if each one in the *unvaccinated* group was PCR negative or not, mainly because many people did not perform the test since they did not have symptoms. Nevertheless, the antibody levels in this group when compared to the PCR (+) group showed several folds less anti-RBD IgG levels, indicating that this group is less likely to be previously infected. The median time since the last positive PCR test in the PCR(+) group was 182 days, indicating that anti-RBD IgG can persist for over 6 months, in accordance with a recent report where those antibodies persisted for around 20 months following a positive PCR test [24].

In this study, samples were collected from participants just before getting either the first, second, or booster doses, this led to variability in the time of sample collection after vaccination, either due to participants not adhering to the proposed dosing schedule or getting infected in between doses causing an extension of the period until the next dose. However, we found that the time after vaccination had a minimal effect on anti-RBD IgG and IgA levels as indicated by the weak negative correlation between the two, additionally, the comparison between the BBIBP-CorV or BNT162b2 clearly showed that the 2DP group had almost twice as much anti-RBD IgG than 2DS, although the time since the latest vaccine dose in the 2DP and 2DS groups was 113.4 days and 72.46 days, respectively.

Examination of anti-RBD IgM revealed no significant differences between any of the groups tested, probably due to the time at which samples were collected. Anti-RBD IgM levels are significantly decreased at 6 months postinfection [25], in addition, vaccination does not elicit the formation of anti-RBD IgM as natural infection [26], both reasons account for our finding of no significant difference in anti-RBD IgM levels. This study also indicated that anti-RBD IgG but not IgM was important in complement activation following vaccination, where C5b-9 strongly and significantly correlated to the amount of bound C1q, which in turn strongly correlated to the amount of bound anti-RBD-IgG. Though C1q levels correlated to IgA levels as well, it could have been due to the simultaneous increase in IgG and IgA after vaccination at the time of sample collection [27]. Anti-RBD IgG and IgA levels correlated to MBL as well, but this study found no correlation between complement terminal pathway activation and MBL binding.

The binding of the Fc portion of IgG to C1q and subsequent complement activation has been shown to increase the neutralizing ability of antibodies in several types of infections [28–30]. The findings of this study indicated that anti-RBD IgG formed following vaccination with BNT162b2 and BBIBP-CorV bound C1q, as shown by the strong and significant correlation between the two in both vaccine groups. In addition to activating complement, binding of C1q to antibodies can improve virus neutralization in other less recognized mechanisms, for example, C1q binding to antibodies against West Nile virus was shown to reduce the stoichiometric requirements for neutralization [29], indicating that C1q binding on its own, even without subsequent complement activation can enhance neutralization.

A similar mechanism for pathogen neutralization without complement activation could be at play with regard to the MBL binding we demonstrated in this study. To our knowledge, binding of MBL to vaccine-induced antibodies was not demonstrated before, but MBL interaction with the glycans of antibodies is well described in the literature, especially in the case of polymeric IgA [31] and agalactosylated glycoforms of IgG [32].

We believe that the neutralizing ability of antibodies formed against SARS-COV-2 should be investigated in the presence and absence of C1q, MBL, and other complement components, especially in light of advancements that allow the manipulation of the Fc portion of monoclonal antibodies to enhance effector functions [33], a process that is relevant in vaccine production as well [34].

When examining C1q binding in different vaccine groups, data indicated that serum from participants who took one or two doses of BNT162b2 bound more C1q than serum from participants who took one or two doses of BBIBP-CorV. While the ratio C1q/anti-RBD IgG did not differ significantly between the two vaccine types, the ratio C5b-9/C1q did. This finding could be explained by the difference in C1q binding stability in the presence of IgG oligomers, indeed, several studies reported on the enhanced ability of IgG oligomers, especially hexamers, in binding C1q and initiating complement activation [35, 36]. Notably, the formation of IgG oligomers and subsequent complement binding and activation depends to some extent on the nature of the IgG, in addition to antigen distribution on the surface [35, 37].

It was recently shown that anti-RBD IgG from SARS-COV-2 infected individuals can bind C1q and activate complement, and the amount of complement activation was associated with disease severity [38]. In our study, bound C1q and formed C5b-9 were both higher in the PCR(+) compared to the *unvaccinated* group but did not reach statistical significance, probably since participants were not hospitalized and did not have an active infection at the time of sample collection. Another recent study investigated Fc glycosylation patterns in IgG formed following BNT162b2 vaccination or natural infection, and found a unique proinflammatory Fc composition following BNT162b2 vaccination [20]. Such data emphasizes the importance of identifying unique effector functions in antibodies formed following vaccination and how they relate to the protection offered by the vaccine.

The main limitation of this study is that while it investigated IgG, IgA, and IgM levels, which together account for over 98% of serum antibodies, it did not investigate IgE or IgD levels, nor did it investigate the subclasses of IgG that were responsible for the observed complement activation. Nevertheless, previous studies into IgG subclasses indicate that IgG1 and IgG3 were the predominant subclasses formed after both vaccinations with BNT162b2 and natural infection [20]. A similar study of an inactivated vaccine against COVID-19 (CoronaVac) indicated that IgG1 and IgG3 were the most prevalent in serum following two doses [39]. Those subclasses, especially IgG3, have also been reported to be the best in activating complement [40], so it could indicate that they were the responsible subclasses in this study as well.

In conclusion, this study demonstrated increased C1q binding to anti-RBD IgG with subsequent complement activation in individuals receiving one, two, or a booster dose of BNT162b2, compared to individuals who receive one or two doses of BBIBP-CorV. Further studies are required to elucidate the relationship between the neutralizing ability of antibodies formed following COVID-19 vaccination and their ability to bind C1q and activate complement and whether the observed binding of MBL in this study has a role in neutralization in ways other than complement activation.

## Figures and Tables

**Table 1:** Participants' demographics and adverse events following vaccination.

Variables/groups	Unvaccinated (*n* = 22)	Unvaccinated previously PCR+ (*n* = 13)	BNT162b2 one dose (*n* = 29)	BBIBP-CorV one dose (*n* = 21)	BNT162b2 two doses (*n* = 27)	BBIBP-CorV two doses (*n* = 24)	BNT162b2 booster dose (*n* = 15)
Age in years	25.18 (19.96, 30.41)	26.62 (18.67, 34.56)	28.14 (23.34, 32.94)	32.62 (26.55, 38.69)	29.96 (25.29, 34.63)	25.83 (23.01, 28.66)	23.67 (21.49, 25.84)

Gender							
Males	45%	8%	52%	38%	63%	62%	20%
Females	55%	92%	48%	62%	37%	38%	80%

Height in centimeters	167.4 (163.4, 171.3)	164.7 (160.1, 169.2)	169.7 (166.3, 173.1)	167.2 (164.1, 170.4)	172.7 (168.7, 176.6)	173.3 (168.9, 177.6)	166.8 (162.6, 171.0)

Weight in kilograms	67.80 (61.34, 74.26)	66.31 (54.01, 78.60)	69.85 (64.07, 75.64)	67.00 (62.93, 71.07)	74.31 (66.73, 81.88)	72.25 (65.36, 79.14)	59.27 (55.05, 63.48)

Time since the latest infection or vaccine dose in days	NA	187.1 (133.3, 240.9)	34.34 (23.66, 45.03)	102.7 (82.87, 122.5)	113.4 (80.46, 146.4)	72.46 (60.47, 84.45)	67.07 (29.83, 104.3)

Local symptoms after vaccination (1) Pain (2) Swelling (3) Redness (4) Itch	NA	NA	(1) 73% (2) 19% (3) 8% (4) 15%	(1) 31% (2) 6% (3) 0% (4) 6%	(1) 90% (2) 0% (3) 0% (4) 10%	(1) 58% (2) 0% (3) 0% (4) 0%	(1) 87% (2) 0% (3) 7% (4)0%

Systemic symptoms after vaccination (1) Fever (2) Fatigue (3) Headache (4) Chills (5) Vomiting (6) Diarrhea (7) Muscle pain (8) Joint pain	NA	NA	(1) 23% (2) 42% (3) 29% (4) 4% (5) 0% (6) 0% (7) 42% (8) 12%	(1) 13% (2) 31% (3) 13% (4) 6% (5) 0% (6) 0% (7) 13% (8) 13%	(1) 25% (2) 65% (3) 30% (4) 10% (5) 0% (6) 5% (7) 45% (8) 35%	(1) 17% (2) 8% (3) 17% (4) 8% (5) 0% (6) 0% (7) 8% (8) 8%	(1) 33% (2) 40% (3) 27% (4) 27% (5) 0% (6) 0% (7) 20% (8) 13%

^a^Data is presented either as mean and (95% confidence intervals) or as a percent of participants.

## Data Availability

The data used to support the findings of this study are included within the article.

## Supplementary Materials

Supplementary Materials Supplementary Figure 1: anti-RBD Immunoglobulins and their correlation to the time of sample collection. Serum samples were collected from participants at various time points after vaccination and incubated with RBD-coated plates; subsequently, HRP-tagged anti-human IgG, IgA, or IgM antibodies were used to detect the presence of bound IgG, IgA, and IgM, respectively. (S/N) represents the chemiluminescence signal generated from wells incubated with 1% serum divided by the signal in control wells incubated with PBST. (A), (B), and (C) are scatter plots that correlate anti-RBD IgG, IgA, and IgM levels, respectively, to the time since the last vaccine dose (in days) in all vaccinated participants. Spearman's correlation coefficient (*r*_s_), *P* value, and the number of participants (*n*) are displayed on the plot. Supplementary Figure 2: measurement of C3d formation. Participants were divided by their vaccination status, type of vaccine, and dose of vaccine. Serum samples were incubated with RBD-coated plates; the formation of C3d was subsequently measured using an indirect immunoassay. (S/N) represents the chemiluminescence signal generated from wells incubated with 1% serum divided by the signal in control wells incubated with PBST. Each circle represents one participant, bold horizontal lines represent the mean of each group, while whiskers represent the standard deviation. Some error bars were clipped at the axis limit. Only significant pairwise comparisons are displayed, ^∗^*P* ≤ 0.05, ^∗∗^*P* ≤ 0.01, ^∗∗∗∗^*P* ≤ 0.0001.

## References

[1] Sikora D., Rzymski P. (2022). COVID-19 vaccination and rates of infections, hospitalizations, ICU admissions, and deaths in the European economic area during autumn 2021 wave of SARS-CoV-2. *Vaccine*.

[2] Ismail AlHosani F., Eduardo Stanciole A., Aden B. (2022). Impact of the Sinopharm's BBIBP-CorV vaccine in preventing hospital admissions and death in infected vaccinees: results from a retrospective study in the emirate of Abu Dhabi, United Arab Emirates (UAE). *Vaccine*.

[3] Tartof S. Y., Slezak J. M., Fischer H. (2021). Effectiveness of mRNA BNT162b2 COVID-19 vaccine up to 6 months in a large integrated health system in the USA: a retrospective cohort study. *The Lancet*.

[4] Vályi-Nagy I., Matula Z., Gönczi M. (2021). Comparison of antibody and T cell responses elicited by BBIBP-CorV (Sinopharm) and BNT162b2 (Pfizer-BioNTech) vaccines against SARS-CoV-2 in healthy adult humans. *Geroscience*.

[5] Sadarangani M., Marchant A., Kollmann T. R. (2021). Immunological mechanisms of vaccine-induced protection against COVID-19 in humans. *Nature Reviews Immunology*.

[6] Hartley G. E., Edwards E. S. J., Aui P. M. (2020). Rapid generation of durable B cell memory to SARS-CoV-2 spike and nucleocapsid proteins in COVID-19 and convalescence. *Science Immunology*.

[7] Salvatori G., Luberto L., Maffei M. (2020). SARS-CoV-2 SPIKE PROTEIN: an optimal immunological target for vaccines. *Journal of Translational Medicine*.

[8] Tai W., He L., Zhang X. (2020). Characterization of the receptor-binding domain (RBD) of 2019 novel coronavirus: implication for development of RBD protein as a viral attachment inhibitor and vaccine. *Cellular molecular immunology*.

[9] Walls A. C., Park Y. J., Tortorici M. A., Wall A., McGuire A. T., Veesler D. (2020). Structure, function, and antigenicity of the SARS-CoV-2 spike glycoprotein. *Cell*.

[10] Suthar M. S., Zimmerman M. G., Kauffman R. C. (2020). Rapid generation of neutralizing antibody responses in COVID-19 patients. *Cell Reports Medicine*.

[11] Bournazos S., Gupta A., Ravetch J. V. (2020). The role of IgG fc receptors in antibody-dependent enhancement. *Nature Reviews Immunology*.

[12] Wen J., Cheng Y., Ling R. (2020). Antibody-dependent enhancement of coronavirus. *International Journal of Infectious Diseases*.

[13] Sim R. B., Tsiftsoglou S. A. (2004). Proteases of the complement system. *Biochemical Society Transactions*.

[14] Carroll M. V., Sim R. B. (2011). Complement in health and disease. *Advanced Drug Delivery Reviews*.

[15] Carroll M. C., Isenman D. E. (2012). Regulation of humoral immunity by complement. *Immunity*.

[16] Java A., Apicelli A. J., Liszewski M. K. (2020). The complement system in COVID-19: friend and foe?. *JCI Insight*.

[17] Polycarpou A., Howard M., Farrar C. A. (2020). Rationale for targeting complement in COVID-19. *EMBO Molecular Medicine*.

[18] Noris M., Benigni A., Remuzzi G. (2020). The case of complement activation in COVID-19 multiorgan impact. *Kidney International*.

[19] Varghese P. M., Tsolaki A. G., Yasmin H. (2020). Host-pathogen interaction in COVID-19: pathogenesis, potential therapeutics and vaccination strategies. *Immunobiology*.

[20] Farkash I., Feferman T., Cohen-Saban N. (2021). Anti-SARS-CoV-2 antibodies elicited by COVID-19 mRNA vaccine exhibit a unique glycosylation pattern. *Cell Reports*.

[21] Fisher J., Sørensen O. E., Abu-Humaidan A. H. A. (2020). Using chemiluminescence imaging of cells (CLIC) for relative protein quantification. *Scientific Reports*.

[22] Schindelin J., Arganda-Carreras I., Frise E. (2012). Fiji: an open-source platform for biological-image analysis. *Nature Methods*.

[23] Dunn O. J. (1964). Multiple comparisons using rank sums. *Technometrics*.

[24] Alejo J. L., Mitchell J., Chang A. (2022). Prevalence and durability of SARS-CoV-2 antibodies among unvaccinated US adults by history of COVID-19. *JAMA*.

[25] Marcotte H., Piralla A., Zuo F. (2022). Immunity to SARS-CoV-2 up to 15 months after infection. *iScience*.

[26] Ruggiero A., Piubelli C., Calciano L. (2022). SARS-CoV-2 vaccination elicits unconventional IgM specific responses in naive and previously COVID-19-infected individuals. *eBioMedicine*.

[27] Zurac S., Nichita L., Mateescu B. (2021). COVID-19 vaccination and IgG and IgA antibody dynamics in healthcare workers. *Molecular Medicine Reports*.

[28] Feng J. Q., Mozdzanowska K., Gerhard W. (2002). Complement component C1q enhances the biological activity of influenza virus hemagglutinin-specific antibodies depending on their fine antigen specificity and heavy-chain isotype. *Journal of Virology*.

[29] Mehlhop E., Nelson S., Jost C. A. (2009). Complement protein C1q reduces the stoichiometric threshold for antibody- mediated neutralization of west Nile virus. *Cell Host & Microbe*.

[30] Thielens N. M., Tacnet-Delorme P., Arlaud G. J. (2002). Interaction of C1q and Mannan-binding lectin with viruses. *Immunobiology*.

[31] Roos A., Bouwman L. H., van Gijlswijk-Janssen D. J., Faber-Krol M. C., Stahl G. L., Daha M. R. (2001). Human IgA activates the complement system via the Mannan-binding lectin pathway. *The Journal of Immunology*.

[32] Arnold J. N., Dwek R. A., Rudd P. M., Sim R. B. (2006). Mannan binding lectin and its interaction with immunoglobulins in health and in disease. *Immunology Letters*.

[33] Gunn B. M., Bai S. (2021). Building a better antibody through the fc: advances and challenges in harnessing antibody fc effector functions for antiviral protection. *Human Vaccines & Immunotherapeutics*.

[34] Nawab D. H. (2021). Vaccinal antibodies: fc antibody engineering to improve the antiviral antibody response and induce vaccine-like effects. *Human Vaccines & Immunotherapeutics*.

[35] Wang G., de Jong R. N., van den Bremer E. T. J. (2016). Molecular basis of assembly and activation of complement component C1 in complex with immunoglobulin G1 and antigen. *Molecular Cell*.

[36] Zwarthoff Seline A., Widmer K., Kuipers A. (2021). C1q binding to surface-bound IgG is stabilized by C1r2s2 proteases. *Proceedings of the National Academy of Sciences*.

[37] Strasser J., de Jong R. N., Beurskens F. J. (2019). Unraveling the macromolecular pathways of IgG oligomerization and complement activation on antigenic surfaces. *Nano Letters*.

[38] Jarlhelt I., Nielsen S. K., Jahn C. X. H. (2021). SARS-CoV-2 antibodies mediate complement and cellular driven inflammation. *Frontiers in Immunology*.

[39] Chen W., Zhang L., Li J. (2022). The kinetics of IgG subclasses and contributions to neutralizing activity against SARS-CoV-2 wild-type strain and variants in healthy adults immunized with inactivated vaccine. *Immunology*.

[40] Vidarsson G., Dekkers G., Rispens T., Rispens T. (2014). IgG subclasses and allotypes: from structure to effector functions. *Frontiers in Immunology*.

