# Supplementary material for: Anti-SARS-Cov-2 S-RBD IgG Formed after BNT162b2 Vaccination Can Bind C1q and Activate Complement
Source: J Immunol Res. 2022 Dec 17;2022:7263740. doi: 10.1155/2022/7263740 (PMC9789906; doi:10.1155/2022/7263740)
Supplement: Supplementary Materials — Supplementary Figure 1: anti-RBD Immunoglobulins and their correlation to the time of sample collection. Serum samples were collected from participants at various time points after vaccination and incubated with RBD-coated plates; subsequently, HRP-tagged anti-human IgG, IgA, or IgM antibodies were used to detect the presence of bound IgG, IgA, and IgM, respectively. (S/N) represents the chemiluminescence signal generated from wells incubated with 1% serum divided by the signal in control wells incubated with PBST. (A), (B), and (C) are scatter plots that correlate anti-RBD IgG, IgA, and IgM levels, respectively, to the time since the last vaccine dose (in days) in all vaccinated participants. Spearman's correlation coefficient (rs), P value, and the number of participants (n) are displayed on the plot. Supplementary Figure 2: measurement of C3d formation. Participants were divided by their vaccination status, type of vaccine, and dose of vaccine. Serum samples were incubated with RBD-coated plates; the formation of C3d was subsequently measured using an indirect immunoassay. (S/N) represents the chemiluminescence signal generated from wells incubated with 1% serum divided by the signal in control wells incubated with PBST. Each circle represents one participant, bold horizontal lines represent the mean of each group, while whiskers represent the standard deviation. Some error bars were clipped at the axis limit. Only significant pairwise comparisons are displayed, ∗P ≤ 0.05, ∗∗P ≤ 0.01, ∗∗∗∗P ≤ 0.0001. [file 7263740.f1.pdf]

Supplementary material

Anti-SARS-Cov-2 S-RBD IgG formed after BNT162b2 vaccination can bind C1q and activate complement

Anas H. A. Abu-Humaidan[1*], Fatima M. Ahmad[1,2], Dima Awajan[3], Raba'a F. Jarrar[1], Nader Alaridah[1]

[1] Department of Pathology, Microbiology and Forensic Medicine, School of Medicine, The University of Jordan, Amman 11942, Jordan.

[2] Department of the Clinical Laboratory Sciences, School of Science, The University of Jordan, Amman 11942, Jordan.

[3] Department of Clinical Pharmacy and Therapeutics, Applied Science Private University,

Amman 11931, Jordan

*Address correspondence to:
Anas Abu-Humaidan M.D. Ph.D.
E-mail: A.abuhumaidan@ju.edu.jo
Tel. number: +962779227922

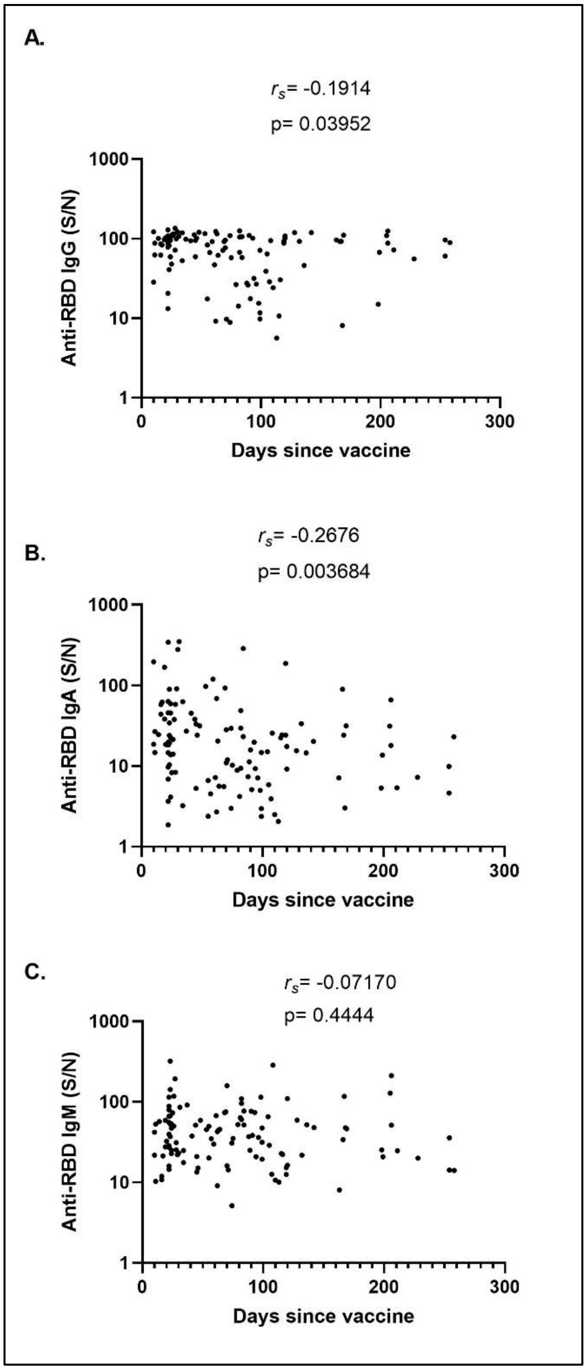

Supplementary Figure 1. Anti-RBD Immunoglobulins and their correlation to the time of sample collection. Serum samples were collected from participants at various time points after vaccination and incubated with RBD-coated plates; subsequently, HRP-tagged anti-human IgG, IgA, or IgM antibodies were used to detect the presence of bound IgG, IgA, and IgM, respectively. (S/N) represents the chemiluminescence signal generated from wells incubated with 1% serum divided by the signal in control wells incubated with PBST. (A), (B), and (C) are scatter plots that correlate anti-RBD IgG, IgA, and IgM levels, respectively to the time since the last vaccine dose (in days) in all vaccinated participants. Spearman's correlation coefficient (r_s), p-value, and the number of participants (n) are displayed on the plot.

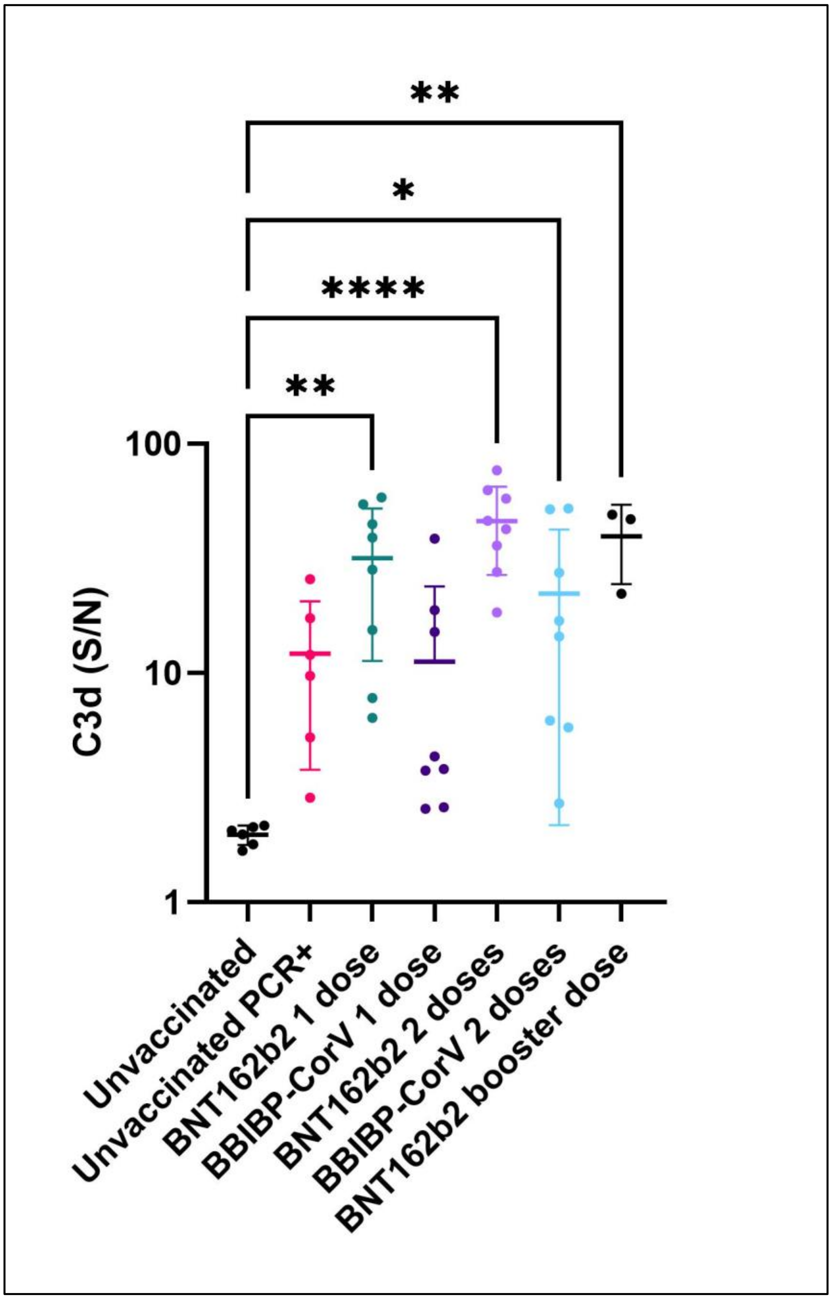

Supplementary Figure 2. Measurement of C3d formation. Participants were divided by their vaccination status, type of vaccine, and dose of vaccine. Serum samples were incubated with RBD-coated plates; the formation of C3d was subsequently measured using an indirect immunoassay. (S/N) represents the chemiluminescence signal generated from wells incubated with 1% serum divided by the signal in control wells incubated with PBST. Each circle represents one participant, bold horizontal lines represent the mean of each group, while whiskers represent the standard deviation. Some error bars were clipped at the axis limit. Only significant pairwise comparisons are displayed, * $P \leq 0.05$, ** $P \leq 0.01$, **** $P \leq 0.0001$.
